# Population genomic analyses of schistosome parasites highlight critical challenges facing endgame elimination efforts

**DOI:** 10.1038/s41598-021-86287-y

**Published:** 2021-03-25

**Authors:** Jonathan A. Shortt, Laura E. Timm, Nicole R. Hales, Zachary L. Nikolakis, Drew R. Schield, Blair W. Perry, Yang Liu, Bo Zhong, Todd A. Castoe, Elizabeth J. Carlton, David D. Pollock

**Affiliations:** 1 Department of Biochemistry and Molecular Genetics, University of Colorado Anschutz Medical Campus, Aurora, CO 80045 USA; 2 Colorado Center for Personalized Medicine, University of Colorado Anschutz Medical Campus, Aurora, CO 80045 USA; 3 Department of Biology, University of Texas at Arlington, Arlington, TX 76109 USA; 4 Department of Ecology and Evolutionary Biology, University of Colorado Boulder, Boulder, CO 80309 USA; 5 Institute of Parasitic Disease, Sichuan Center for Disease Control and Prevention, Chengdu, People’s Republic of China; 6 Department of Environmental and Occupational Health, University of Colorado Anschutz Medical Campus, Colorado School of Public Health, Aurora, CO 80045 USA

**Keywords:** Population genetics, Parasitic infection, Genomics

## Abstract

Schistosomiasis persists in Asian regions despite aggressive elimination measures. To identify factors enabling continued parasite transmission, we performed reduced representation genome sequencing on *Schistosoma japonicum* miracidia collected across multiple years from transmission hotspots in Sichuan, China. We discovered strong geographic structure, suggesting that local, rather than imported, reservoirs are key sources of persistent infections in the region. At the village level, parasites collected after referral for praziquantel treatment are closely related to local pre-treatment populations. Schistosomes within villages are also highly related, suggesting that only a few parasites from a limited number of hosts drive re-infection. The close familial relationships among miracidia from different human hosts also implicate short transmission routes among humans. At the individual host level, genetic evidence indicates that multiple humans retained infections following referral for treatment. Our findings suggest that end-game schistosomiasis control measures should focus on completely extirpating local parasite reservoirs and confirming successful treatment of infected human hosts.

## Introduction

Schistosomiasis is a neglected tropical disease that impacts an estimated 200 million people globally^1–3^ causing fibrosis of the liver and bladder, anemia, and in some species, cancer^1,2,4,5^. Schistosomiasis control programs in China, beginning in the 1950s, are responsible for a 99% reduction in schistosomiasis infection prevalence, with approximately 54,000 infections in China in 2016^6–8^. The modern schistosomiasis control program in China is a multi-pronged strategy including health education, testing and treatment, application of molluscicides to snail habitat, and treatment of bovines^9,10^. While control programs are generally effective^9,10^, transmission hotspots remain for reasons that are not well understood^11,12^. Several regions, including regions outside of China^13^, have experienced re-emergence of schistosomiasis or no further declines in prevalence^11^, and our team, among others, has found high infection rates in recent years^14^. These infections are perplexing partly because they arise in areas where control programs are ongoing and infected snails are not readily identified^10^.

The persistence of infection despite ongoing control measures highlights gaps in our knowledge of the natural history of schistosomes and the epidemiology of schistosome infection. As China continues towards the goal of schistosomiasis elimination by 2025^15^, new insights into factors affecting schistosome transmission are needed. Evolutionary and population genetic studies can yield insights that can be used to fill these gaps and increase the effectiveness of control programs, such as how parasite populations change in response to mass administration of chemotherapy^16^. An in-depth understanding of *S. japonicum* population structure in Sichuan, China—a region currently experiencing schistosomiasis re-emergence despite on-going, aggressive control measures^10^—can provide crucial and actionable insights into how a parasite population on the brink of elimination is able to persist.

Detailed insight into schistosome transmission patterns in response to treatment could inform future control programs implemented in other parts of the world where parasitic helminths are endemic. Most population genetic studies in schistosomes have been limited by the number of loci, small sample sizes, or both^17–21^, and thus provided limited resolution in answering questions about population structure. However, recent advances in genomic technologies are making it possible to address previously inaccessible questions and promise to grant greater insight into the persistence of schistosome infections. Here, we apply a reduced representation genome sequencing approach^22–24^ to sample tens of thousands of single nucleotide polymorphisms (SNPs) from hundreds of miracidia (the offspring of infective schistosome mating pairs) longitudinally collected across nearly a decade. These data provide unprecedented resolution of patterns of schistosome population structure across a geographically small area in Sichuan, China that highlight key features of regional infection hotspots. We further describe an approach to discern between different degrees of relatedness, enabling the inference of source infections using high-resolution genomic data.

## Results

In total, 272 miracidia preserved on FTA indicator cards were sequenced using double digest restriction-site associated DNA sequencing (ddRADseq)^25^. This reduced representation genome sequencing approach was applied following whole genome amplification, and generated a total of 1.8B reads. After filtering sequences for quality, mapping reads to the *S. japonicum* reference genome, and excluding both low-coverage loci (Supplementary Fig. S1) and miracidia with excess missing genotypes, there were 72,797 variable sites in 200 miracidia. The details of the distribution of these miracidia across hosts and villages are provided in Supplementary Table S1. We further filtered out low-confidence SNP calls as missing data, resulting in a final set containing 33,901 variants.

### Population analyses

**Figure 1:**
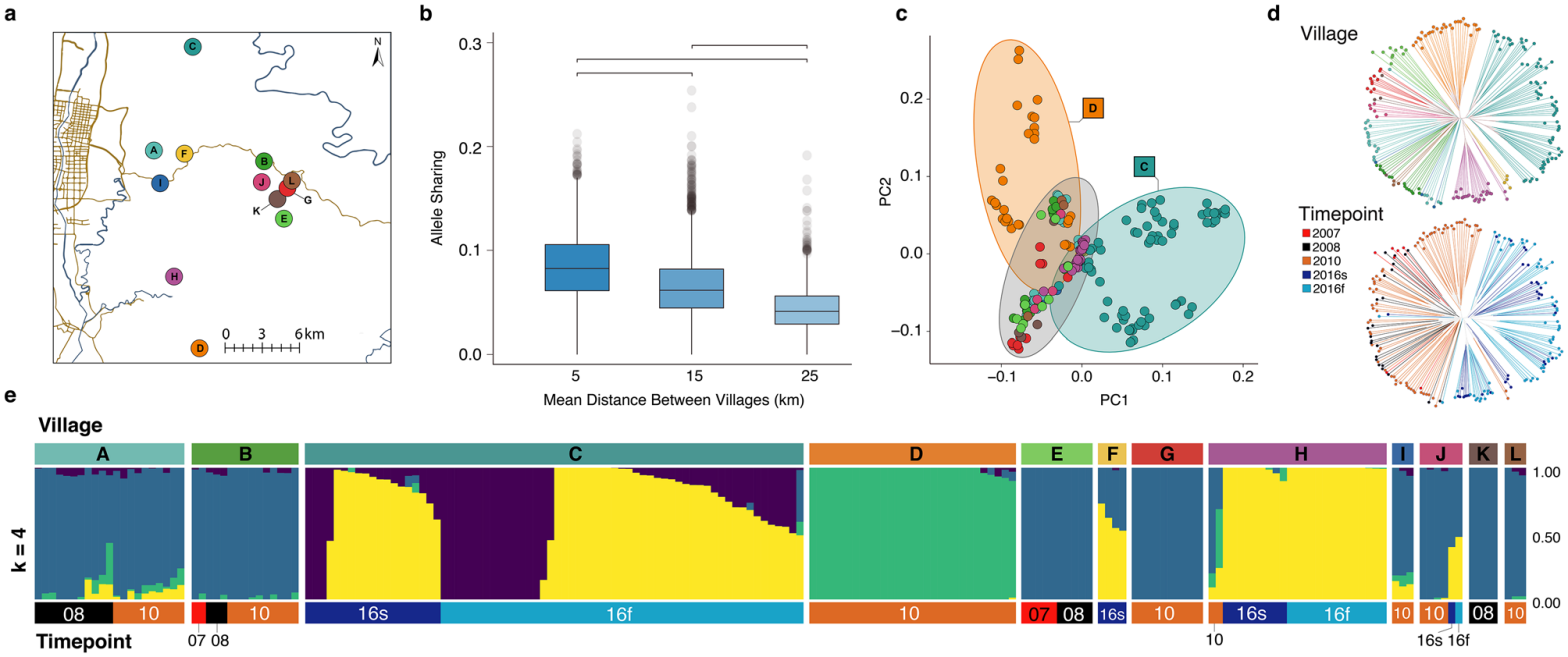
Genetic and geographic structure of *Schistosoma japonicum* miracidia sampled in Sichuan, China. (**a**) Map showing locations of the 12 villages sampled, indicated by colored dots. Yellow lines represent major roads and blue lines indicate rivers and major streams. The map was created with ArcGIS ArcMap^52^ (version 10.6; https://desktop.arcgis.com/en/arcmap/). (**b**) Proportion of rare alleles shared among villages with mean, interquartile ranges, and outliers beyond the 2.5th percentile shown. Inter-village distances are Euclidean. All comparisons were significantly different (all p < 2.2 × 10^–16^; Mann–Whitney *U* test). (**c**) Principal component analysis (PCA) of genetic variation from 200 miracidia across all 12 villages. The first two principal components (PC1 and PC2) respectively account for 4.2% and 2.5% of the genetic variation among individuals. (**d**) Neighbor-joining tree of miracidia colored by village (top) and sampling timepoint (bottom). (**e**) ADMIXTURE plot showing optimal *k* = 4 genetic clusters grouped by village and sampling timepoint. Timepoints are labeled with year of collection (e.g., 2008 in **d** or 08 in **e**); Summer and late Fall 2016 collections are labeled with small s or f, respectively.

To determine whether schistosome infections are acquired from local or regional sources, we evaluated the spatial distribution of schistosome genetic diversity across the study region. Genetic structure indicates that the parasites are more related within villages than between villages, with allele sharing decreasing significantly with geographic distance between villages (Fig. 1a,b, Supplementary Fig. S2). Population structure is strong enough that most villages have a unique, discernible population of miracidia. For example, the first two principal components in principal component analysis (PCA) of genetic variation across samples distinguish miracidia in the two most distant villages, C (the northernmost village sampled in this study) and D (the southernmost village), from other villages (Fig. 1c); additional principal components separate most other villages into clear clusters based on genetic similarity (Supplementary Fig. S3). Phylogenetic analysis of miracidia also clusters villages, with most villages occupying their own clade (Fig. 1d, Supplementary Fig. S4). This trend was not seen when neighbor-joining trees were labeled by timepoint (Fig. 1d). Estimates of population structure using *ADMIXTURE*^26^ support this finding and identify further substructure within villages, particularly village C (Fig. 1e).

The genetic structure of schistosomes within villages indicates that local infection sources were not fully eliminated by whole-village praziquantel treatments between sampling points. Miracidia from the same village fall into characteristic *ADMIXTURE* clusters regardless of sampling timepoint (Fig. 1e, villages A, B, C, E, and H; see also Supplementary Figs. S4, S5), and miracidia collected from the same timepoint fall into multiple clades on the phylogenetic tree (Fig. 1d). However, there is a notable difference in genetic structure in village J (within the eastern cluster of villages) between 2010 and 2016, the largest time span present in the data (Fig. 1d,e). While structure appears to be retained over time in many cases, our ability to conduct longitudinal sampling for every village was limited and the extent to which population structure is conserved is variable. Our resolution is also limited by limited sampling of hosts in particular villages, with some villages represented by a single individual host.

To confirm that broad patterns in our results were robust when sibling miracidia were removed, a sibling-pruned dataset was generated and analyzed in the same way as the full dataset. Results of these analyses were indeed qualitatively similar to those based on the full dataset and are presented in Supplementary Figs. S6–S10.

### Identification of family clusters and relatedness estimates

**Figure 2:**
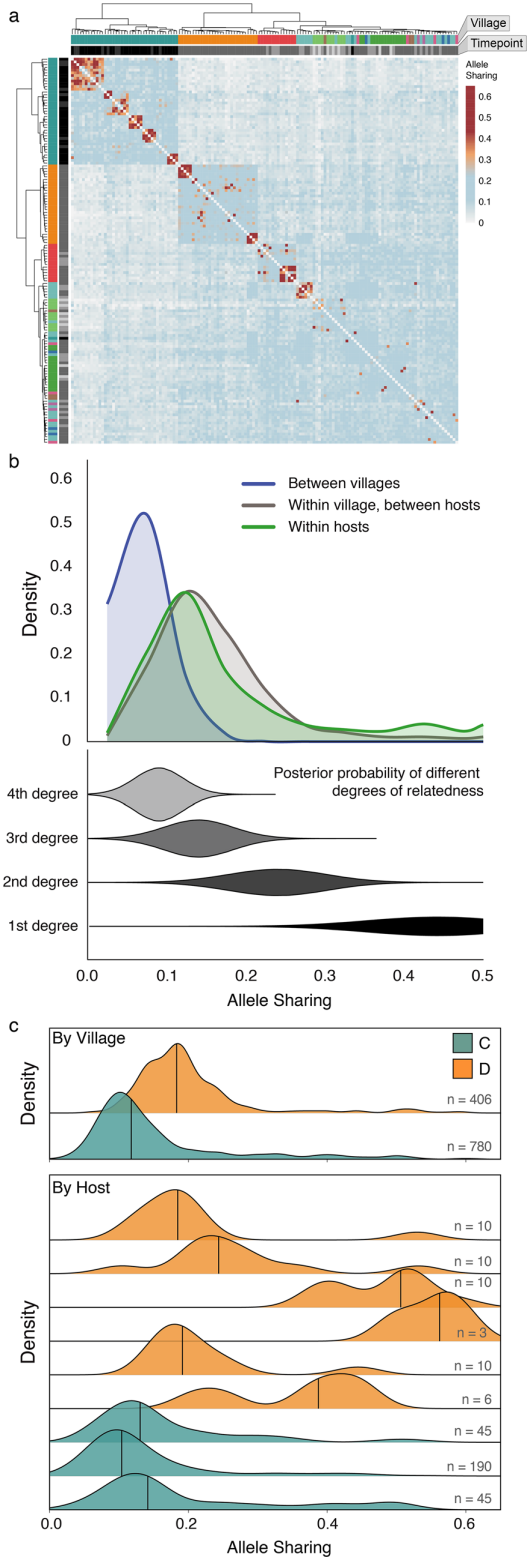
Genetic relatedness of *Schistosoma japonicum* miracidia within and between villages. (**a**) Heatmap of allele sharing between all sampled *Schistosoma japonicum* miracidia. Rows and columns are ordered using hierarchical clustering and annotated with village and timepoint. (**b**) Distributions are shown for allele sharing between miracidium pairs sampled from different villages (blue), within villages but different hosts (grey), and within hosts (green). The posterior probabilities for different degrees of relatedness are indicated by width for 1st to 4th degree relatives in the lower plot. (**c**) Distributions of within-village (top panel) and within-host (lower panel) allele sharing are shown for villages C (green) and D (orange). The total number of comparisons underlying each distribution is shown on the right.

**Figure 3:**
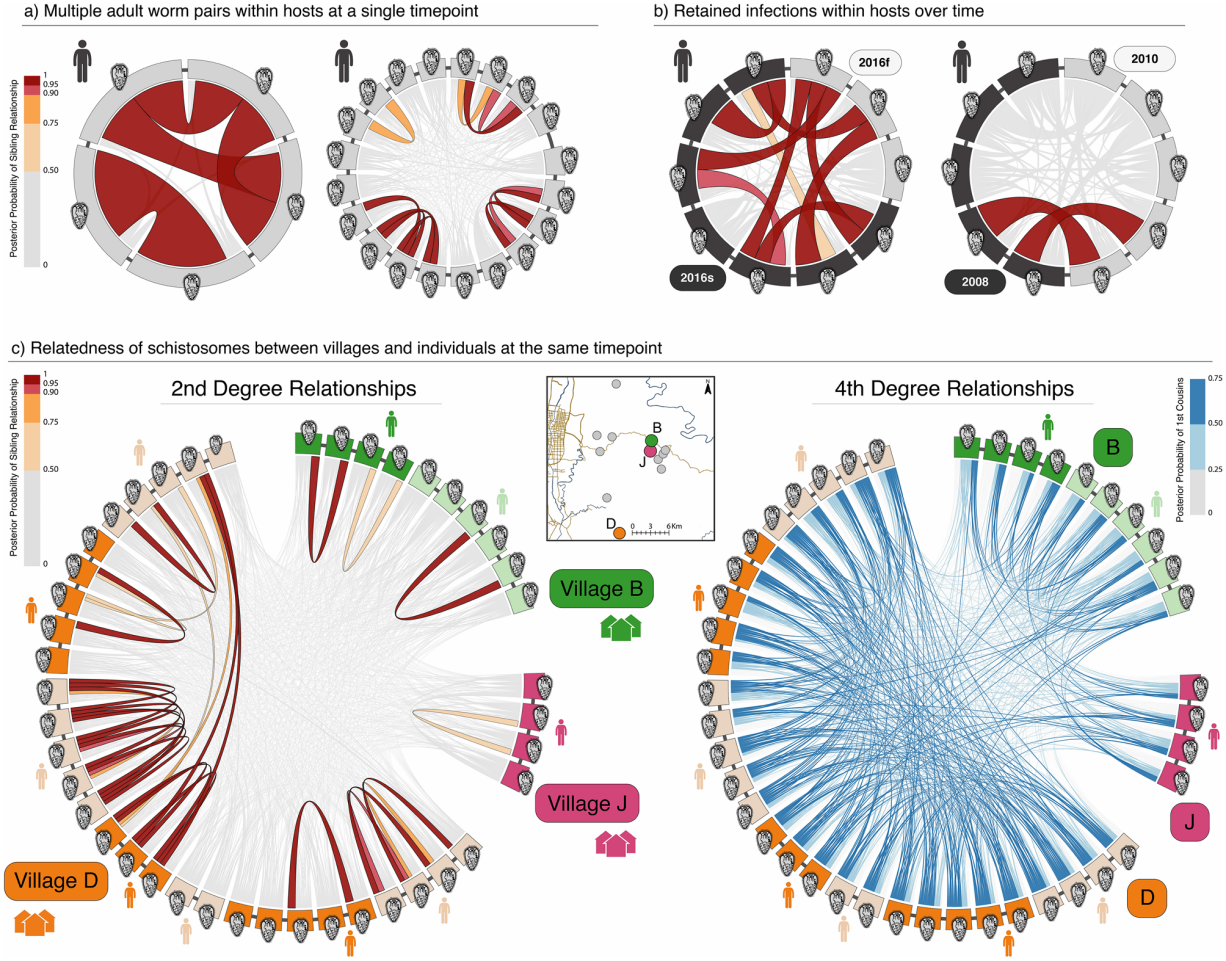
Relatedness of *Schistosoma japonicum* miracidia within and between hosts. Examples highlight relatedness structures indicating multiple infections, retained infections, evidence for clones, and inbreeding within villages. Hosts are indicated with human figures, with different miracidia collected from a single host connected by thin dark grey lines. Ribbons between miracidia show posterior probabilities of degree of relatedness through color (scale to side) and by ribbon width. (**a**) Two examples of multiple sibling clusters (2 and 4) within hosts are shown. In the second example, 8 miracidia are not in sibling clusters (all connections are in light grey), indicating a high multiplicity of infection sources (a minimum of 12 mating pairs) in this host. (**b**) Two examples of likely retained infections over time are inferred from the sibling-level miracidia sampled from the same host at different timepoints separated by five months and two years. (**c**) Miracidia from multiple hosts living in villages B, J, and D and sampled at the same timepoint are shown with gaps between different villages. In the left graph, sibling-level relatedness is shown, and a case of sibling-level relatedness between miracidia across two individuals indicates clonal parents. In the right graph, cousin-level relatedness emphasizes that strongly supported first-cousin relationships are common among miracidia within villages and sparse between villages.

Measures of relatedness among miracidia allow inference of fine-scale transmission patterns. In the absence of reliable allele frequencies and/or robust linkage information, we used the proportion of rare alleles shared between all pairs of miracidia to calculate the posterior probabilities of first-, second-, third-, or fourth-degree relationships between members of a pair (Fig. 2a; Supplementary Table S2). We find evidence that miracidia from the same village tend to be closely related (Figs. 1b and 2b). Posterior probabilities of relatedness calculated from allele sharing (“Methods”) indicate that schistosome first cousins (3rd degree relatives) are extremely common within villages, but much rarer between villages (Fig. 2b). Because we only sample the progeny of adult mating schistosomes, a first-degree relationship between a pair of miracidia indicates that members of the pair are siblings, and as expected, pairs of miracidia collected from the same human host are often siblings (1st degree relatives; Fig. 2b). However, we also find a large number of 2nd degree relatives within villages (Fig. 2c, village D). It seems reasonable that most of these are double first cousins, given the high frequency of first cousins within villages. Separate clutches of parasite siblings were identified within individual human hosts (Fig. 3a), indicating infection by multiple mating pairs. We also found multiple examples of human hosts with sibling clusters that span multiple sampling timepoints (Fig. 3b); while the possibility that a human host was reinfected with clones cannot be discounted, this is preliminary evidence of retained infection despite the host being referred for intervening treatment protocols (Fig. 3b). One instance of a cross-timepoint sibling cluster was sampled in 2016, when the region implemented directly observed treatment (DOT). Infections detected prior to 2016 could have, in principle, been retained due to non-compliance with treatment. However, the retained infection detected after DOT raises questions about the effectiveness of treatment protocols and concerns that human hosts who failed to clear their infections despite drug treatment may have served as sources of new infections to other community members.

High levels of allele sharing within villages (Fig. 3c) indicate that parasite mate choice is often limited to relatives during the reinfection process. This limitation implies that following treatment, infections in a village may have been re-established by a small number of genetically unique schistosomes, likely reflecting the effectiveness of local schistosomiasis control programs^10,27^. However, these results also suggest that long-term elimination may require identification and targeted treatment of remaining local parasite sources. The genetic structure of schistosomes within villages indicates that local infection sources were not fully eliminated by whole-village praziquantel treatments between sampling points. For example, we inferred two possible treatment failures based on the identification of apparent siblings collected from the same host before and after treatment cycles, one of which was sampled at two timepoints in 2016, when treatment was directly observed (Fig. 3b).

The existence of clones could produce false inferences of sibling relationships (and thus retained infections) within individual hosts across timepoints, and cannot be ruled out entirely if the number of cercaria-emitting snails in the environment is small enough that individuals are reinfected with identical worms from the same snails, or if identical juvenile worms residing in the liver survive treatment. We discount this partly due to the ~ 6-month lifespan of infected *Oncomelania* snails^28^ and the months required to develop worms from cercariae and form mating pairs within a human host. Time-separated clonal double infections would require the environmental condition that the individual snails produce clonal cercariae from the time of the initial infection (followed by development, mate pairing, detection, and treatment—a minimum of 40 days^29^) until the time of the second infection. In contrast, the retention of infections over time, due either to non-compliance with treatment or treatment failure, is a more obvious explanation and supported by prior evidence^14^. Furthermore, cross-host siblings indicating clonal pairs are rare (Fig. 3c). Thus, we generally expect that clonality has had little impact on our within-host inferences of retained infection. Instead, the detection of schistosome clones between human hosts suggests that schistosomiasis control efforts have been highly effective in reducing local snail populations required for producing cercariae.

## Discussion

Our results highlight the important role of epidemiological and genomic data to resolve transmission patterns in areas approaching elimination. Three major trends apparently contributed to the persistence of schistosomiasis in the residual transmission hotspots we studied. First, local parasite reservoirs were a major contributor to local re-introduction of schistosome infections. This is demonstrated by the finding that village miracidia are comprised of closely related populations of *S. japonicum* across timepoints, despite prompt referral for treatment of all positive infections and complementary efforts to eliminate schistosomiasis from these villages during the study period. Second, there is apparent retention of infection in individual hosts despite referral for treatment. Strong evidence for this is provided by identification of sibling clusters from the same human host during sampling events separated by seasons or years. Third, the high degree of relatedness of miracidia from different hosts suggests that humans likely participate in maintaining local schistosomiasis reservoirs and amplify local transmission events, although the participation of non-human mammals cannot be excluded.

We find clear evidence for the successful impact of control measures on population dynamics in *S. japonicum*. This result is somewhat different from some studies in *S. mansoni* and *S. haematobium*/*S. bovis* that observed high gene flow among neighboring populations^30–32^. It seems reasonable to suppose that the difference may lie in the long-term, focused, and comprehensive nature of Chinese schistosome control efforts (which have induced extremely low observed snail abundance), as well as the rural, mountainous topography of our study region. We note that it is not possible to draw definitive conclusions about village-wide population structure in five villages where miracidia were collected from a single host (Supplementary Table S1), however based on the observation that population structure in the more densely-sampled villages is generally stronger between villages than within villages, it seems reasonable to expect that denser sampling within these villages would reveal similar patterns.

Evidence of retained individual infections across sampled timepoints in our local study system raises questions about the negative impact of occasional treatment failures on the effectiveness of control measures. Specifically, human hosts who fail to clear their infections may serve as sources of new infections to other humans. The extent to which human vs. non-human mammalian hosts serve as sources of new infections is an extremely important factor for guiding control efforts. If human hosts sometimes fail to clear their infections following treatment and subsequently serve as sources of infection to others, the effectiveness of treatment protocols should be reviewed and improved. We caution that the frequency and causes of retained infection remain uncertain—including the extent to which treatment failure is due to drug resistance^33^, suboptimal dosing^34^, or non-compliance with treatment^35^—and warrant further investigation. Furthermore, *S. japonicum* is a zoonosis and it is difficult to eliminate non-human mammalian hosts as local reservoirs and amplifiers of human infections. Now that the importance of local reservoirs has been established, ongoing sampling efforts will include a variety of such alternate hosts.

**Figure 4:**
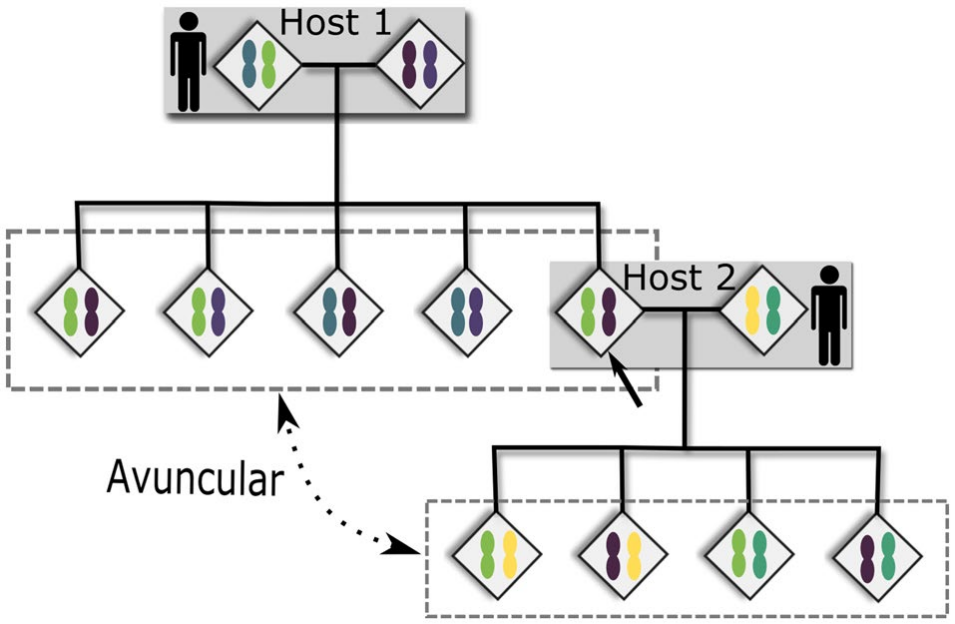
Avuncular relationships among schistosome miracidia. Diamonds indicate schistosomes, with those surrounded by a gray box indicating an adult mating pair, and those surrounded by a dashed box indicating miracidia sibling clutches. Chromosomes within diamonds are colored to indicate different haplotype combinations that could be inherited from parents. The arrow points to a sibling of a miracidia clutch collected from Host 1 that became a parent of another sibling clutch collected from Host 2. This worm is the link that creates the depicted avuncular relationship between the offspring of schistosomes within Host 1 and Host 2.

Furthermore, the evidence for inbreeding among schistosomes complicates the evaluation of short inter-human infection pathways. Such evaluation is also complicated because the human-infective cercaria stage of schistosomes that originates from snails is clonal, and genetically identical cercariae may produce multiple infections in one or more human hosts^36^. The most direct human-to-human infection pathway, involving only a snail intermediate host, would yield avuncular relationships between miracidia from each host (Fig. 4). However, because of inbreeding and clonality, we were unable to differentiate between the types of 2nd degree relations (double first cousins, half-siblings, or avuncular). Due to the high frequency of first-cousin level relationships within villages, we suspect that many, if not most, of the 2nd degree relations observed are double first cousins. Although rare, observations of sibling-level relatives across human hosts demonstrate that clonal infections occurred in our samples (Fig. 3c), and so clonal infections could also explain some 1st and 2nd degree relatives observed between human hosts.

We expect that some of these questions can be resolved by the sampling and acquisition of denser variant information with more loci per Mbp. Increased directed sampling will enable the estimation of key epidemiological parameters such as the frequency of treatment failure, the number of active mating pairs within a human host, and the frequency of clonal infections. Sampling of non-human mammalian hosts can potentially establish a role for such hosts as both reservoirs and amplifiers of re-introduced human infections. It is worth noting that it may not be possible to eliminate a role for non-human hosts. If non-human hosts contribute low-frequency infection rates or if a non-human host type is unidentified, such sources become nearly impossible to detect. Denser variant information, such as that obtained by whole genome sequencing, along with recent improvements in the *S. japonicum* reference genome^37^, will allow construction of extended haplotypes (local linkage groups) that should be able to better distinguish among types of 2nd degree relatives and potentially extend pedigrees. Such definitive inference of infection pathways would allow the establishment of frequencies of transmission routes in the local schistosome re-establishment.

The work presented here exemplifies how population genomic studies can illuminate factors underlying transmission of macroparasites and provide strategic and precise advice to direct control efforts. We find that there are high levels of schistosome inbreeding within villages, that there are consistent, local sources of infection through time, and that some human hosts appear to retain infections despite treatment referral. These findings indicate that the persistence of schistosomiasis in residual transmission hotspots is primarily driven by local transmission and reinfection, with at least some contribution from humans. Based on our findings, end-game control measures should focus on confirmation of schistosome elimination from infected human hosts and complete extirpation of local infection reservoirs.

## Materials and methods

### Miracidia collection and sample selection

Miracidia, the first schistosome larval stage, were collected from 12 villages in Sichuan, China (see Fig. 1a). Infection surveys took place in 2007, 2008, 2010, and in both the summer and fall of 2016. During each survey, village residents submitted fecal samples for three consecutive days and each sample was tested for *S. japonicum* infection using the miracidium hatching test as described in the literature^23^. Individual miracidia were collected from the top of the hatching test flask, rinsed three times in autoclaved, de-ionized water and transferred to Whatman FTA indicator cards using a hematocrit tube or Pasteur pipette drawn to a narrow bore with a flame.

A subset of collected samples were selected for inclusion in the study. This subsampling was designed to include 10–15 miracidia from every village and across multiple timepoints. When possible, we tried to include multiple samples from the same human host and multiple human hosts from each village. However, five villages (E, F, I, K, and L) presented here are represented by multiple miracidia collected from a single host (Supplementary Table S1).

The research involving human subjects was approved by the Sichuan Institutional Review Board, the University of California, Berkeley, Committee for the Protection of Human Subjects, and the Colorado Multiple Institutional Review Board. Participants provided written, informed consent. All experiments were performed in accordance with relevant guidelines and regulations. Anyone testing positive for *Schistosoma japonicum* was informed of their infection status and referred to the local anti-schistosomiasis control station for treatment.

### DNA library preparation and sequencing

DNA library preparation followed a previously published methodology^24^. Briefly, discs containing individual miracidia were excised from Whatman FTA cards using a 2 mm card punch (Whatman WB100029) and DNA from the disc was whole-genome-amplified by isothermal genome amplification, termed “multiple displacement amplification” (MDA), using GenomiPhi v3 (GE Healthcare Biosciences 25660124) amplification tubes with modifications as described in the literature^24^. Amplified DNA was digested for > 8 h with *PstI-HF* and *Sau3AI* at 37 °C followed by a 65 °C heat deactivation step. Following solid phase reverse immobilization (SPRI) DNA extraction, custom adaptors containing an 8-bp unique molecular identifier (UMI) and sequences corresponding to the single-stranded DNA sticky ends generated by digestion and a 6-bp barcode were ligated to digested fragments. Adaptors ligating to *PstI-HF* cuts also contained 6-bp barcodes. Following ligation, sets of 6–8 samples were pooled such that no barcode was used twice within the same pool, and underwent size selection for fragments sizes either 300–600 bp (including adaptor sizes) or 390–690 bp (including adaptor sizes) using a PippinPrep with a 1.5% agarose gel. Following size selection, samples underwent 15 cycles of PCR amplification. Primers used in amplification also contained index sequences and sequences used for Illumina-based sequencing cluster formation (sequences for all adaptors and primers are shown in Supplementary Table S3). Sample pools were then combined in equimolar ratios such that no index sequence was used more than once within each pool. Samples were sequenced on an Illumina HiSeq using v4 chemistry.

### Fastq processing and variant identification

In total, 272 samples were sequenced: 124 samples with 125-bp single end reads each, and 148 samples with 150-bp paired end reads each, resulting in 1,799,089,548 total reads generated. PCR clones were filtered from the reads with the clone filter tool in s*tacks*^38^ using the UMIs contained in each barcode. Sequences were then quality filtered and divided by barcode using the *process_radtags* tool in *stacks*^38^ with restriction enzymes and barcodes supplied as arguments. We ‘rescued’ reads with a single base mutation in the 8-bp barcode or restriction sites (-r). Low-quality reads were removed (-q) to a separate file (-D) and excluded from downstream analysis. On average, 5.61% of reads from each library were identified as clones and removed. An average of 31.37% of reads were filtered from each library due to clonality, ambiguous barcodes/restriction site, or quality, though one library containing barcoded DNA from eight miracidia contained an abnormally high number of reads missing restriction sites in the correct place. This library was retained, with the filters above applied. Excepting this library, an average of 26.96% of total reads were filtered from all reads. Reads passing this series of filters were mapped to the *S. japonicum* reference genome (downloaded from schistodb.net^39,40^) using *bwa mem*^41^. Variants were called from .bam files using *Haplotype Caller* in the Genome Analysis Toolkit^42–44^ with gvcf mode and GenotypeGVCFs. Over 4 million variant sites were found, however most of these sites were sequenced in just one or very few miracidia. Demultiplexed fastq files, as well as bam and bam index files, are available through the NCBI Sequence Read Archive (SRA) database under BioProject PRJNA349754.

### Defining sets of ddRADseq loci

Although most ddRADseq reads mapped reliably to expected ddRADseq loci^24^, loci from off-target reads may add noise to subsequent analyses. To de-noise our data, we identified a set of loci that were reliably recovered at ≥ 20 × depth across the majority of samples in order to retain only those variants that map to ddRADseq loci. Using a custom *perl* script (*cutgenome.pl*; github.com/PollockLaboratory/Schisto), we identified the expected mapping locations of ddRADseq reads in the *S. japonicum* reference genome^40^, with each expected individual ddRADseq locus having two different locations: one for the forward read and one for the reverse read, if applicable.

To identify the subset of these expected loci that could be reliably recovered, we first eliminated miracidia that had fewer than 500K reads post-filtering or less than 20K reads that map to the reference genome with a mapq ≥ 20. We obtained the sequencing depth of each expected ddRADseq locus in each of these 156 remaining ‘high-depth’ miracidia using *bedtools*^45^ intersect. We recorded the coverage of each expected read locus (-c) and required that mapped loci overlapped by at least 50% of an expected read length before incrementing the depth count (-f 0.5). For miracidia that were sequenced with single end sequencing, the cumulative depth of each of the fragment’s possible reads was used as the depth for the locus; for miracidia sequenced with paired end sequencing, the mean depth of the two read loci was used for the fragment’s depth.

From this data set, we identified 9637 expected ddRADseq loci sequenced at ≥ 20 × depth in ≥ 75% of ‘high-depth’ miracidia (see Supplementary Fig. S1). To further restrict variants to the most stringent loci, analyses reported here used only variants from the 6990 expected ddRADseq loci that were close to the target size selection range (170–500 bp). Once these high-confidence loci were identified, they were called across the entire dataset, resulting in reads across 200 samples.

### Variant set creation

Variants then underwent a number of filters as follows: invariant sites were removed, sites with more than two alleles or that contained an indel were removed, and variants that were not within an expected ddRADseq locus were removed. To create our final variant set, we re-coded any sites sequenced at < 10 × coverage as missing data, recoded individual genotypes with GQ < 20 as missing, removed sites that were missing more than 50% of genotype calls, and removed miracidia missing more than 90% of genotype calls (Supplementary Fig. S11). This final filtering resulted in 200 miracidia genotyped at 33,901 sites. The .bed file and .vcfs from different stages of filtering can be downloaded from http://www.EvolutionaryGenomics.com/ProgramsData/SchistoGenomics.

### Population analyses

The parametric tests for population structure we performed require that the provided loci be in linkage equilibrium, however missing genetic distances between neighboring sites and the currently highly fragmented reference genome makes linkage pruning difficult. This problem is compounded in our dataset because a large proportion of the miracidia were suspected to be highly related, which could inflate linkage estimates between sites. Here, we outline the steps we performed to obtain a set of variants likely to be unlinked (though we note that this pruning does not guarantee that all sites used are in linkage equilibrium).

We first identified miracidia that are expected to be closely related by identifying clusters of miracidia that share a proportion of rare alleles greater than 0.45 (see “Identification of family clusters and relatedness estimates” below) between each pair of miracidia, and removed all but one miracidium from each cluster. A total of 83 miracidia remained following this step (see “Identification of family clusters and relatedness estimates” for details). We then pruned linked variants in this putative unrelated set using *plink*’s –indep-pairwise command (v1.90b4.6)^46^ with arguments 1000 100 0.1, which greedily prunes variants with r^2^ > 0.1 from overlapping windows consisting of 1000 variants. Linkage pruning in this way reduced the number of variants in the putatively unrelated set to 6642.

We used *ADMIXTURE*^26^ and these putatively unlinked variants with all 200 miracidia to determine the proportion of each miracidium’s genome that can be attributed to one of *k* different populations. We tested *k* = 2–10, with ten replicates for each *k* and default cross-validation to determine the *k* with the lowest cross validation error (Supplementary Fig. S5).

Principal component analysis (PCA), as implemented in *R*’s (version 3.5.1)^47^ ‘SNPrelate’ package^48^, was applied to the full variant set to assess how genotype differences between miracidia contribute to region-wide variability between samples and villages.

Using all variants, we calculated pairwise genetic distances between miracidia through the distance-based *bitwise.dist* function implemented in *R*’s ‘adegenet’ package^49,50^ and used distances to construct a neighbor-joining tree using the *R*’s ‘ape’ package^51^.

### Identification of family clusters and relatedness estimates

To identify highly related samples in the absence of reliable allele frequency estimates, we used pairwise comparison of shared rare alleles. Rare alleles were defined as alleles whose minor allele frequency ≤ 0.1. Rare allele sharing was calculated between all pairs of samples using only rare variants and a custom *perl* script (findSibClusters.pl; github.com/PollockLaboratory/Schisto) following

1 $$P_{ij} = \frac{1}{L}\mathop \sum \limits_{k = 1}^{L} x_{ijk}$$

where

2 $$x_{ijk} = \left\{ {\begin{array}{*{20}l} 1 & {\quad {\text{if}}\; i\;{\text{ and}}\; j\;{\text{ have}}\;{\text{ the}}\; {\text{same}}\;{\text{ genotype}}\;{\text{ at}}\;{\text{ locus}}\; k} \\ {0.5} & {\quad {\text{if}}\; i\;{\text{ and}}\; j\;{\text{ share}}\;{\text{ one}}\;{\text{ allele}}\;{\text{ at}}\;{\text{ locus}}\; k} \\ 0 & {\quad {\text{if}}\; i\;{\text{ and}}\; j\;{\text{ share}}\;{\text{ no}}\;{\text{ alleles}}\;{\text{ at}}\;{\text{ locus}}\; k} \\ \end{array} } \right.$$

and $P_{ij}$ is the proportion of shared alleles between individuals *i* and *j*, *L* is the number of loci tested, and *k* is a locus for which both individuals *i* and *j* have non-missing genotype calls and individual *i* has a rare variant. To avoid overestimating relationships because of linked variants, we use the mean proportion of rare alleles shared generated from 30 replicates of randomly sampling 2000 loci with replacement for each pairwise comparison. We identified clusters of highly related miracidia such that each miracidium in a cluster shared ≥ 0.45 of its rare alleles with at least one other miracidium in the cluster. Removal of all but one miracidium from each putative sibling cluster (117 individuals) resulted in a data set of 83 miracidia. The sibling-pruned vcf file, which was used to prune linked variants, is available at http://www.EvolutionaryGenomics.com/ProgramsData/SchistoGenomics.

### Calculating posterior probabilities across degrees of relatedness

To generate posterior probability distributions for each degree of relatedness, we first estimated mean levels of unrelated allele sharing, $\hat{\mu }_{unrelated} = 0.04$, as the average pairwise rare allele sharing between individuals from the most geographically distant villages (*n* = 35.6 km) in the full dataset of 200 miracidia. As analysis of pairwise rare allele sharing by inter-village distance indicated a statistically significant decrease in pairwise rare allele sharing as inter-village distance increased, this was determined to be the best, data-driven estimate. To estimate allele sharing among sibling miracidia, we began by identifying clusters of miracidia most likely to be siblings (1st degree relatives): clusters of 3 or more miracidia from the same host and collection timepoint, all with pairwise rare allele sharing proportionn ≥ 0.30 (45 miracidia in 13 clusters). There were an additional 8 pairs of miracidia within the same host that are likely siblings but not part of a big enough cluster. The estimated mean, $\hat{\mu }_{sibs} = 0.44$ and variance, $\hat{\sigma }_{sibs}^{2} = 0.30$, of allele sharing were calculated from eligible pairs (*n* = 60). For intermediate degrees of relatedness, means ($\hat{\mu }_{degree}$) were estimated by successively halving the distance from sibs to unrelated, and variances ($\hat{\sigma }_{degree}^{2}$) were estimated by successively halving the sibling variance for each further degree of relatedness, which will have had twice the number of meioses (e.g., $\hat{\mu }_{2^\circ } = \frac{{(\hat{\mu }_{unrelated} + \hat{\mu }_{sibs} )}}{2}$ and $\hat{\sigma }_{2^\circ }^{2} = \frac{{\hat{\sigma }_{sibs}^{2} }}{2}$). Posterior probabilities were calculated roughly assuming even prior probabilities for each categorical degree of relatedness from siblings to 5th degree relatives and unrelated, and assuming that allele sharing probabilities for each degree of relatedness were distributed normally, i.e., $\sim N(\hat{\mu }_{degree} ,\;\hat{\sigma }_{degree}^{2} )$, a reasonable large-sample approximation.

### Analysis of non-sibling miracidia

As a safeguard against making conclusions about population structure using data that may violate assumptions of independence between samples, we used the posterior probabilities of relatedness to identify sibling clusters (see “Identification of family clusters and relatedness estimates” and “Calculating posterior probabilities across degrees of relatedness”) and generated a sibling-pruned dataset that includes 81 non-sibling miracidia. We used the sibling-pruned dataset to repeat analyses described in “Population analyses”, namely: ADMIXTURE, PCA, and construction of a neighbor-joining tree. Finally, we subset the rare-allele sharing described in “Identification of family clusters and relatedness estimates” to include only the 81 non-sibling miracidia and compared the proportions of shared rare alleles between all pairs of remaining miracidia to the distance between the two villages where members of the pair were collected.

## Supplementary Information

Supplementary Figures.

Supplementary Table 1.

Supplementary Table 2.

Supplementary Table 3.

## Data Availability

Sequences generated during this work have been deposited in the NCBI Sequence Read Archive under BioProject PRJNA349754. The vcf file used in analysis is available at http://www.EvolutionaryGenomics.com/ProgramsData/SchistoGenomics and custom scripts are available at github.com/PollockLaboratory/Schisto.
