# Supplementary material for: Population genomic analyses of schistosome parasites highlight critical challenges facing endgame elimination efforts
Source: Sci Rep. 2021 Mar 25;11:6884. doi: 10.1038/s41598-021-86287-y (PMC7994584; doi:10.1038/s41598-021-86287-y)
Supplement: Supplementary file 1 — Supplementary Figures. [file 41598_2021_86287_MOESM1_ESM.pdf]

Supplementary Materials for

Population genomic analyses of schistosome parasites highlight critical challenges facing endgame elimination efforts

Jonathan A. Shortt, Laura E. Timm, Nicole R. Hales, Zachary L. Nikolakis, Drew R. Schield,

Blair W. Perry, Yang Liu, Bo Zhong, Todd A. Castoe, Elizabeth J. Carlton, and David D.

Pollock

Correspondence to: David.Pollock@CUAnschutz.edu (DDP)

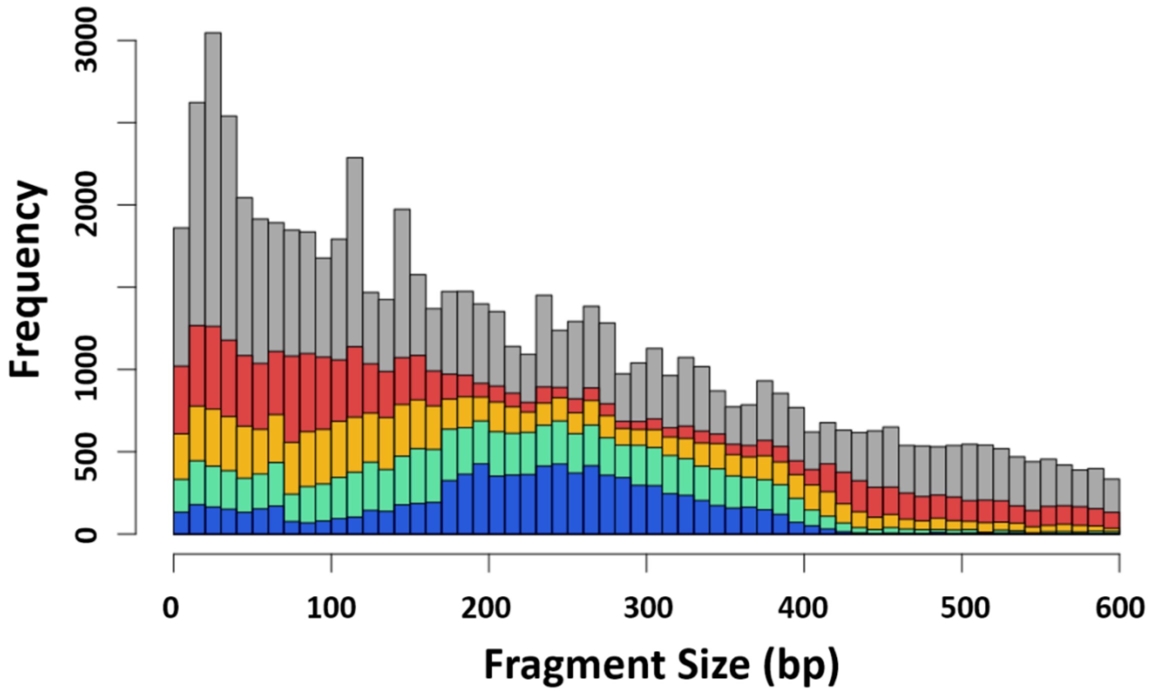

Supplementary Fig. S1.

Size distribution of expected ddRADseq loci. Size distribution of expected ddRADseq loci sequenced in at least 75% of 'high-depth' miracidia (see text) at different depths. Gray = all ddRAD fragments, red = 2x coverage, orange = 5x coverage, green = 10x coverage, blue = 20x coverage.

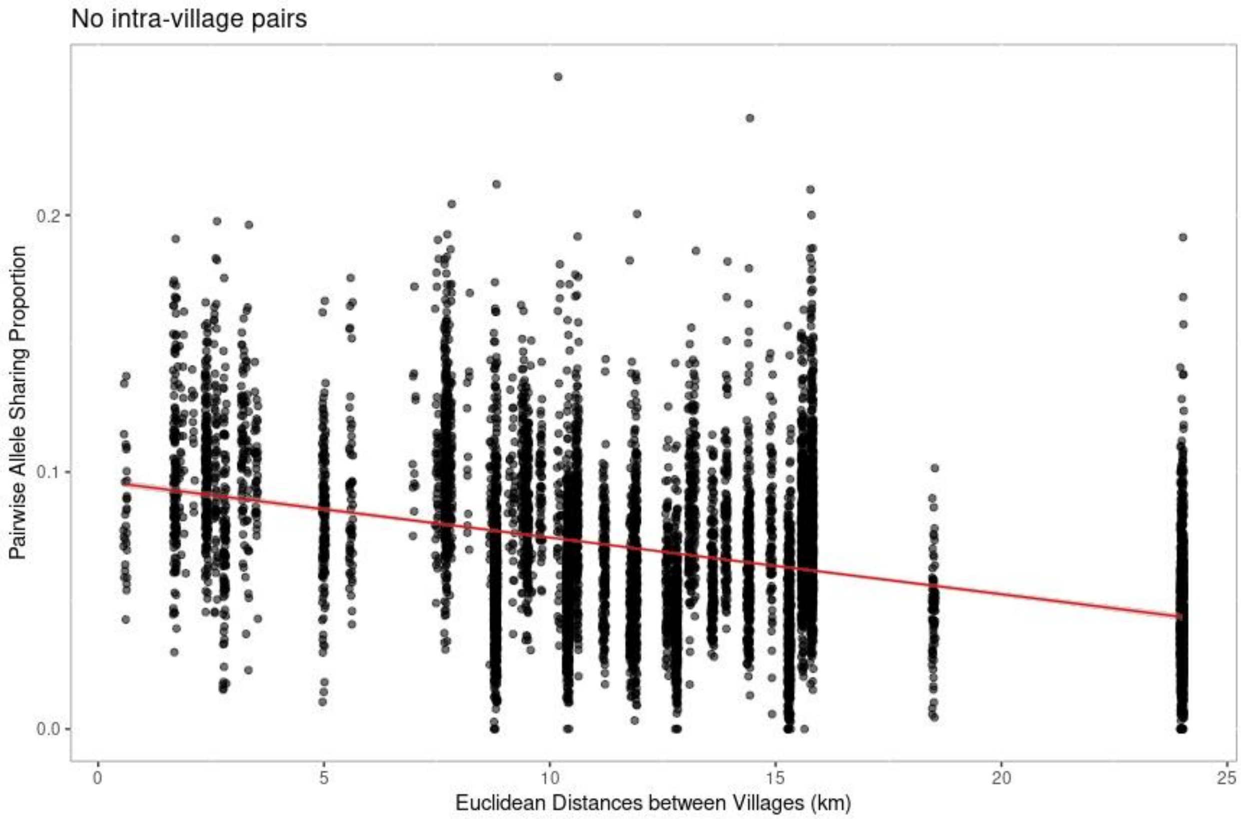

Supplementary Fig. S2.

Proportion of rare alleles shared between pairs of miracidia by distance between the villages where miracidia were collected. Proportions are not shown for pairs of miracidia collected from the same village.

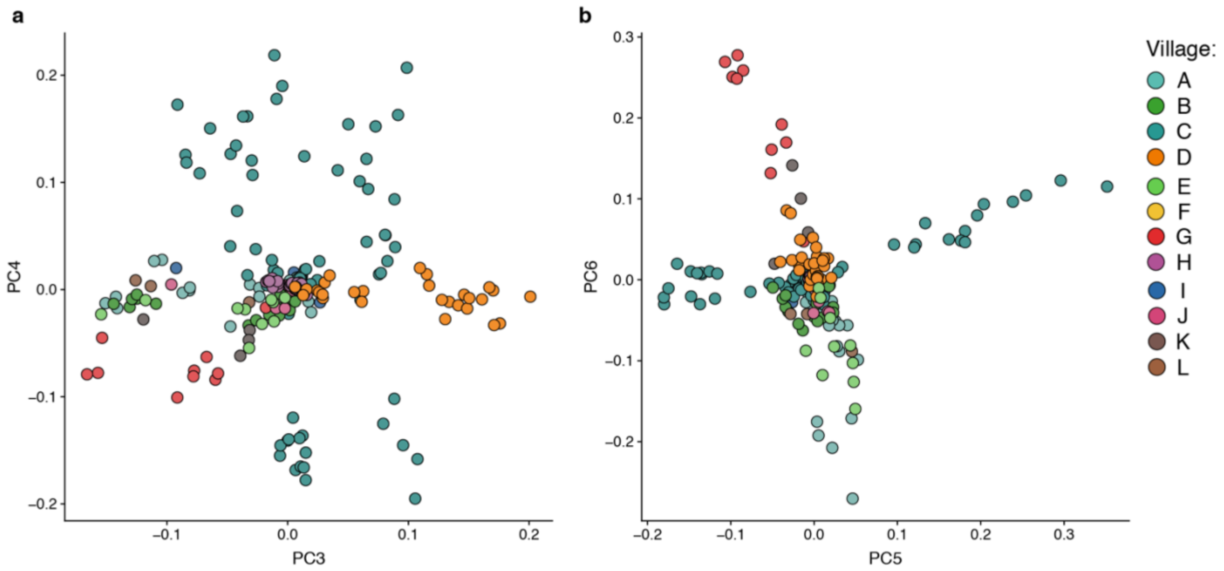

Supplementary Fig. S3.

Principal components analysis of 200 *Schistosoma japonicum* miracidia from 12 villages. Each point corresponds to an individual miracidium, with colors of the points indicating the village from which the miracidium was collected. PCs 3 and 4 (which explain 2.22% and 1.99% of variation, respectively) are shown in the figure on the left and PCs 5 and 6 (1.67% and 1.64%, respectively) are shown in the figure on the right. Village colors correspond to those shown in Fig 1a.

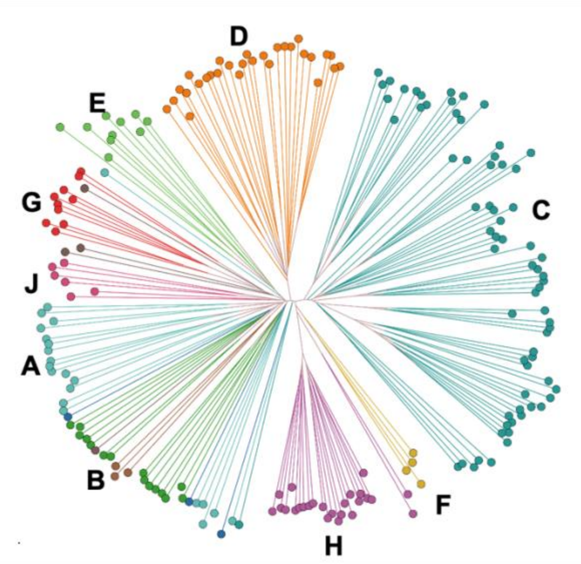

Supplementary Fig. S4.

Neighbor-joining tree of miracidia colored by village. Labels near clusters from a single village indicate the village from which miracidia were collected. Colors are the same as in Figure 1a from the main text.

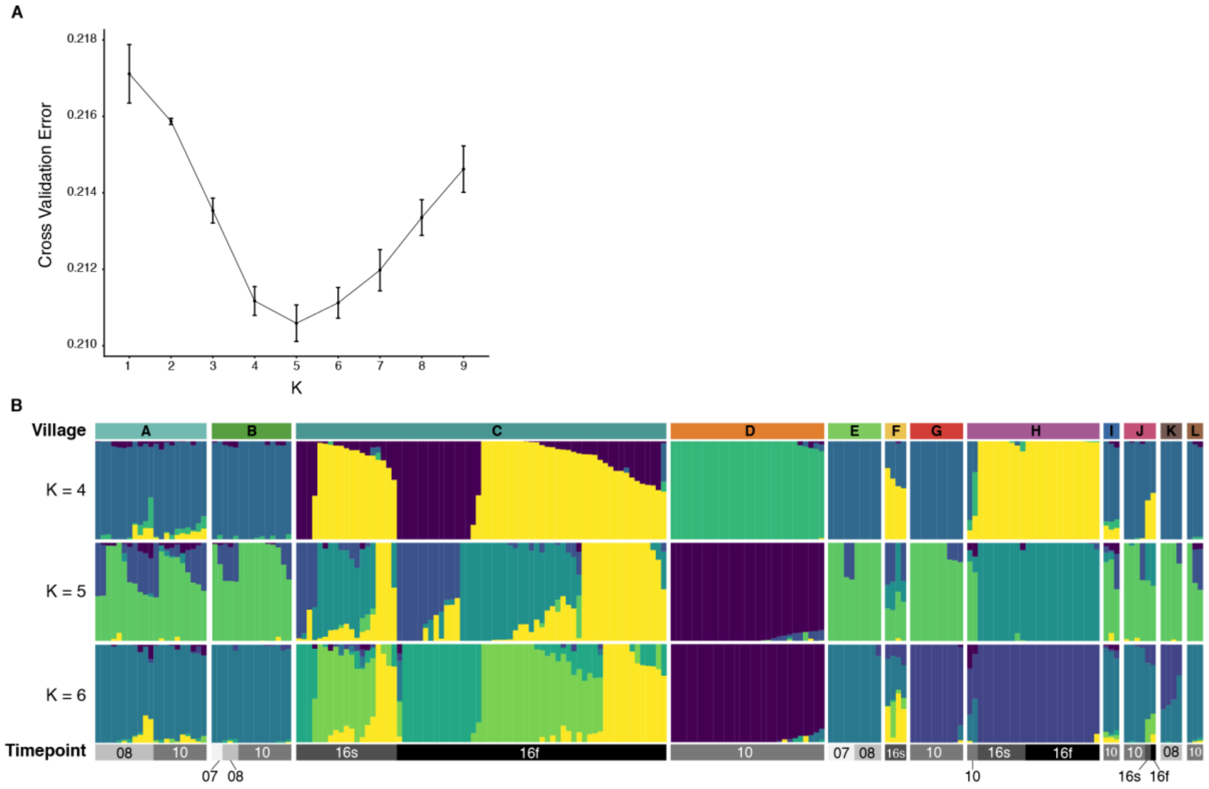

Supplementary Fig. S5.

Supplemental *ADMIXTURE* plots. As in Fig 1e, the *ADMIXTURE* plots show genetic clusters grouped by village and sampling timepoint. A) Cross-validation error for different values *k* based on 10 runs at each *k*. Error bars represent standard error of the mean for each cross-validation value at the specified *k*. B) Graphical results for *k*=4, 5, and 6 for comparison. *k*=4 was shown in figure 1.

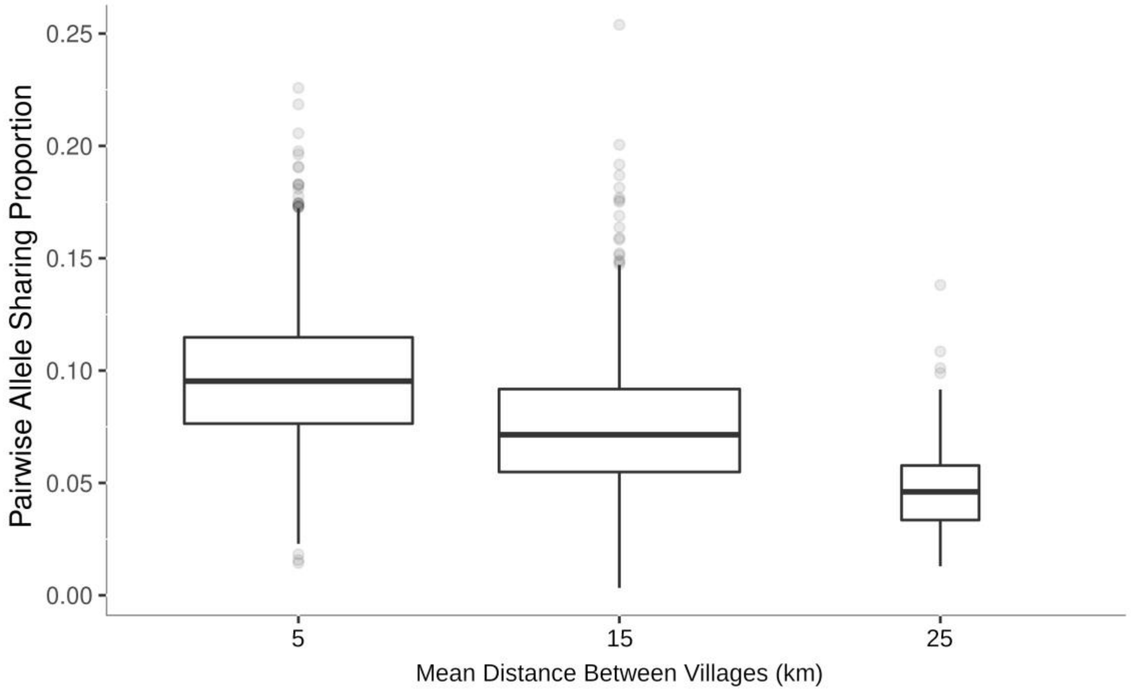

Supplementary Fig. S6.

Proportion of rare alleles shared between non-sibling miracidia among villages with mean, interquartile ranges, and outliers beyond the 2.5th percentile shown. Inter-village distances are Euclidean. All comparisons were significantly different.

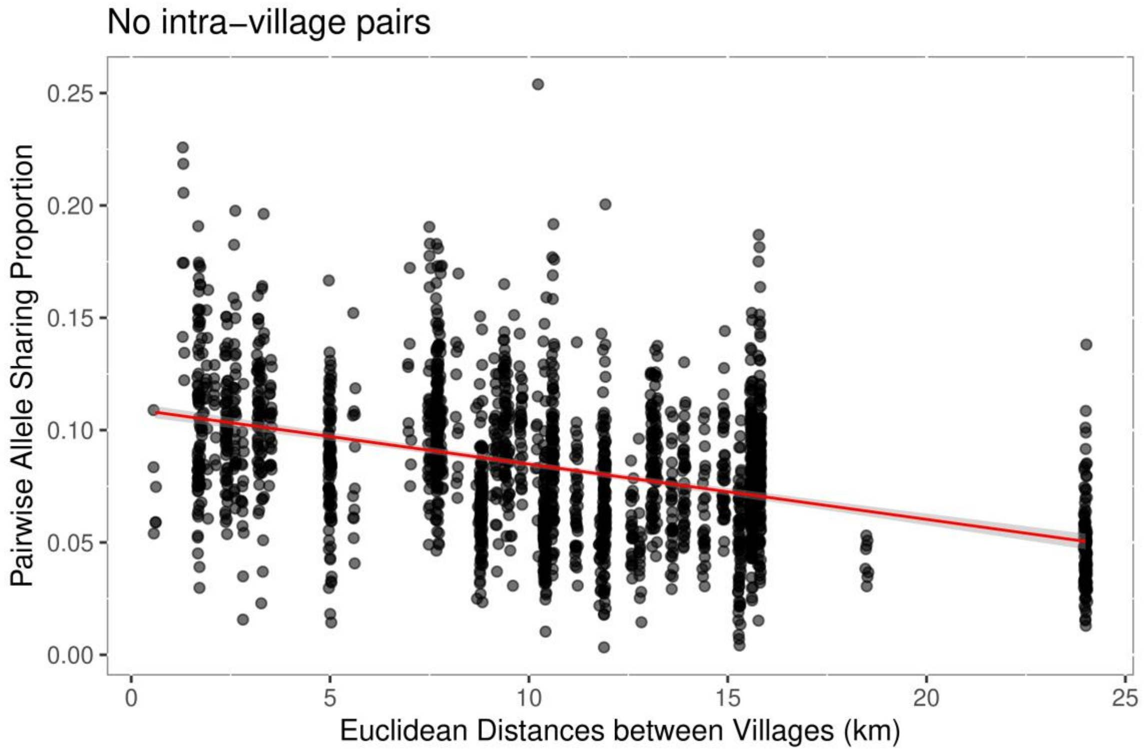

Supplementary Fig. S7.
Proportion of rare alleles shared between pairs of non-sibling miracidia by distance between the villages where miracidia were collected. Proportions are not shown for pairs of miracidia collected from the same village.

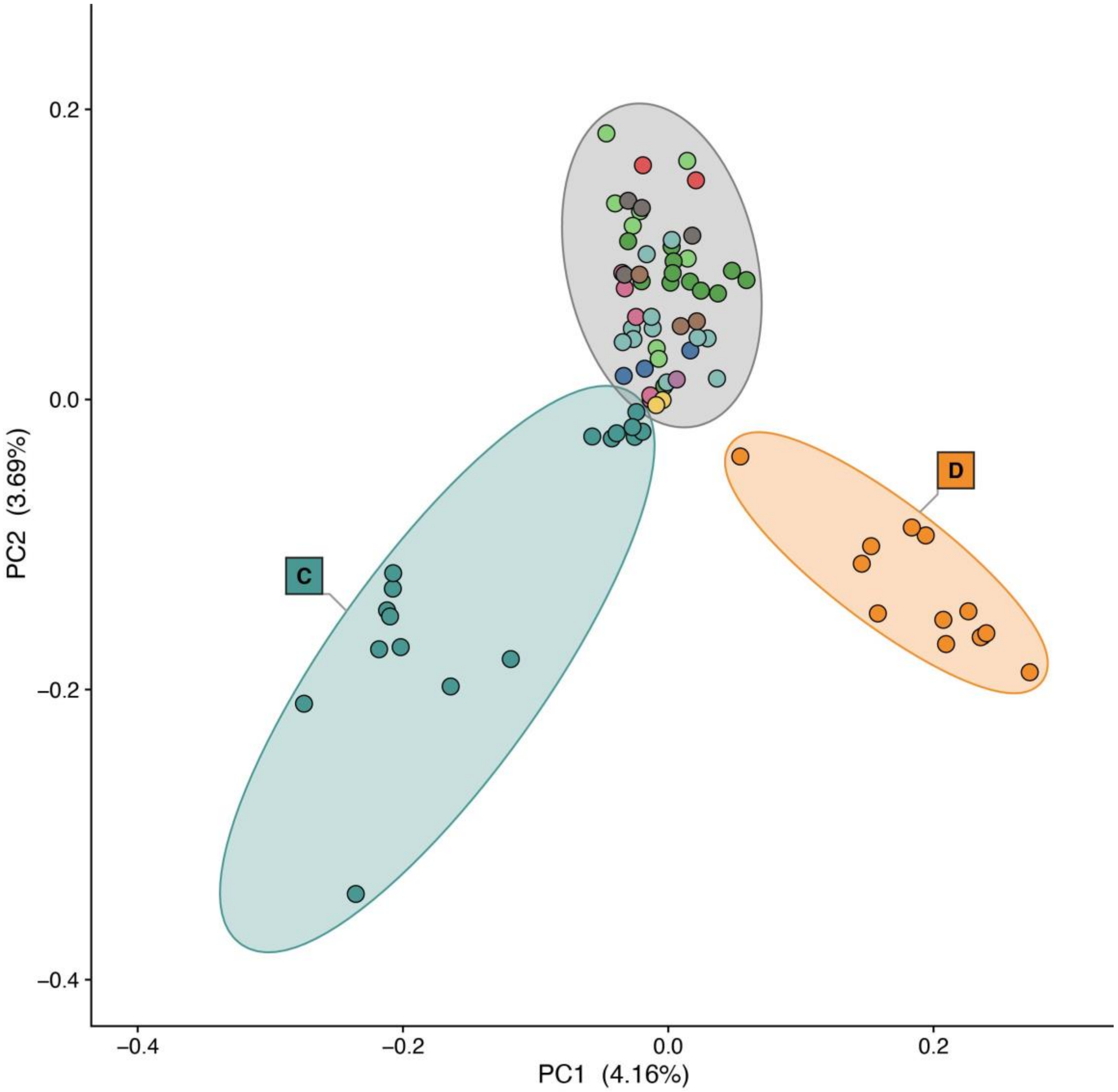

Supplementary Fig. S8.

Principal components analysis of 81, non-sibling *Schistosoma japonicum* miracidia. Each point corresponds to an individual miracidium, with colors of the points indicating the village from which the miracidium was collected. The first two principal components (PC1 and PC2) respectively account for 4.16% and 3.69%. Village colors correspond to those shown in Fig 1a.

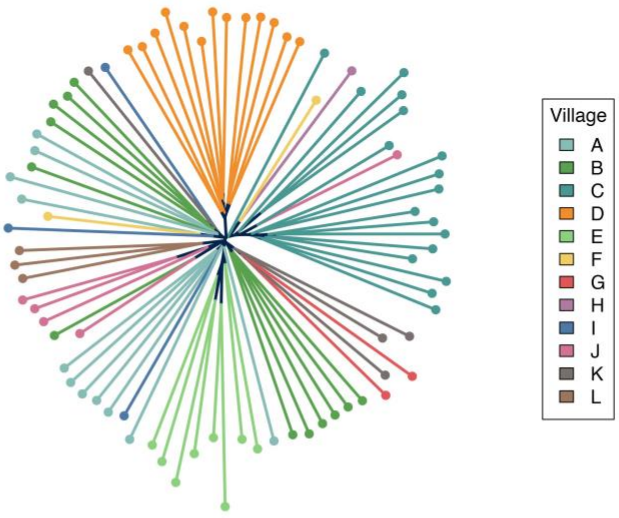

Supplementary Fig. S9.
Neighbor-joining tree of non-sibling miracidia colored by village. Colors are the same as in Figure 1a from the main text.

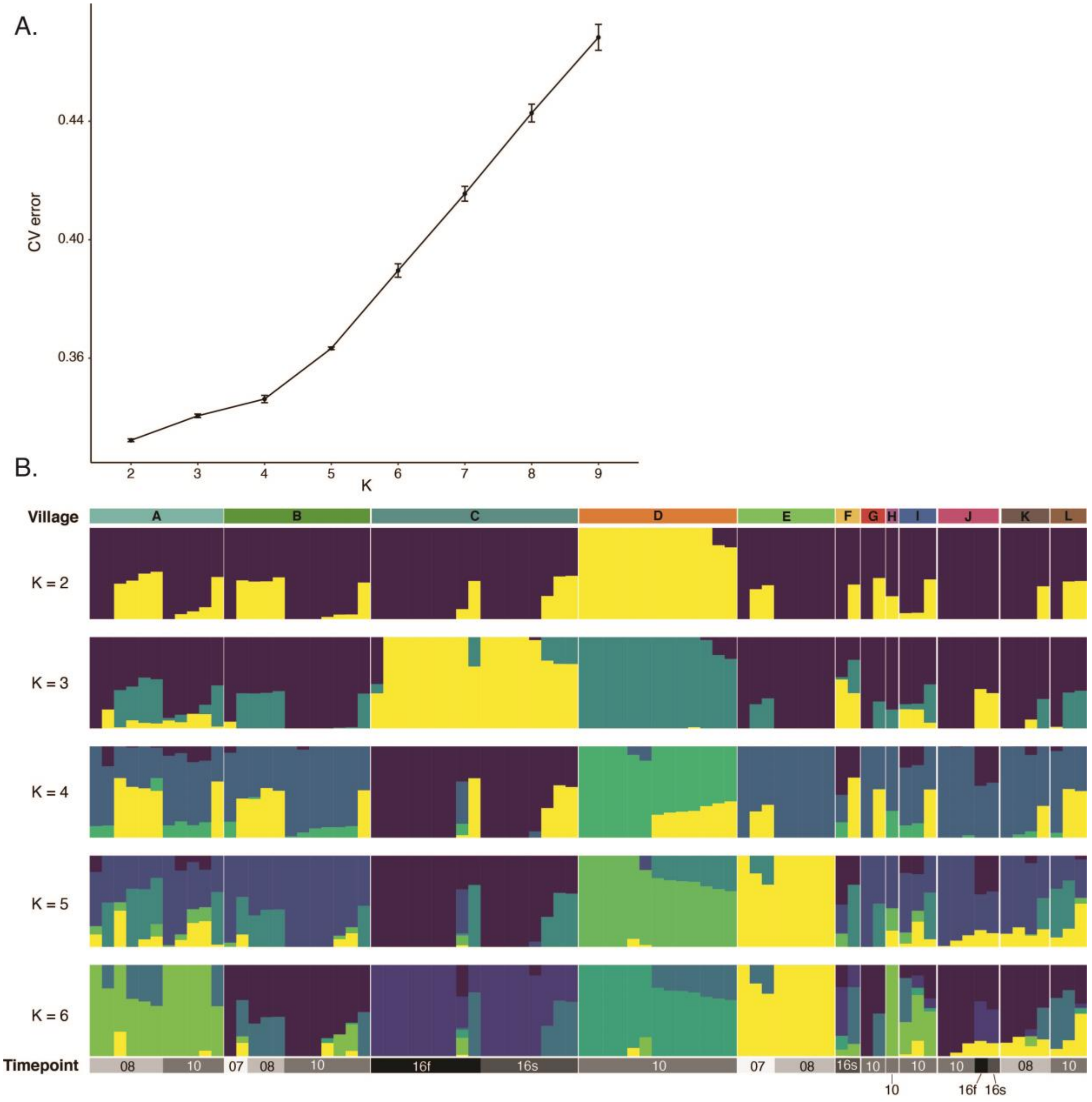

Supplementary Fig. S10.
Supplemental *ADMIXTURE* plots analyzing the sibling-pruned dataset of 81 miracidia. As in Fig 1e, the *ADMIXTURE* plots show genetic clusters grouped by village and sampling timepoint. A) Cross-validation error for different values of *k* based on 10 runs at each *k*. Error bars represent standard error of the mean for each cross-validation value at the specified *k*. B) Graphical results for *k*=2, 3, 4, 5, and 6.

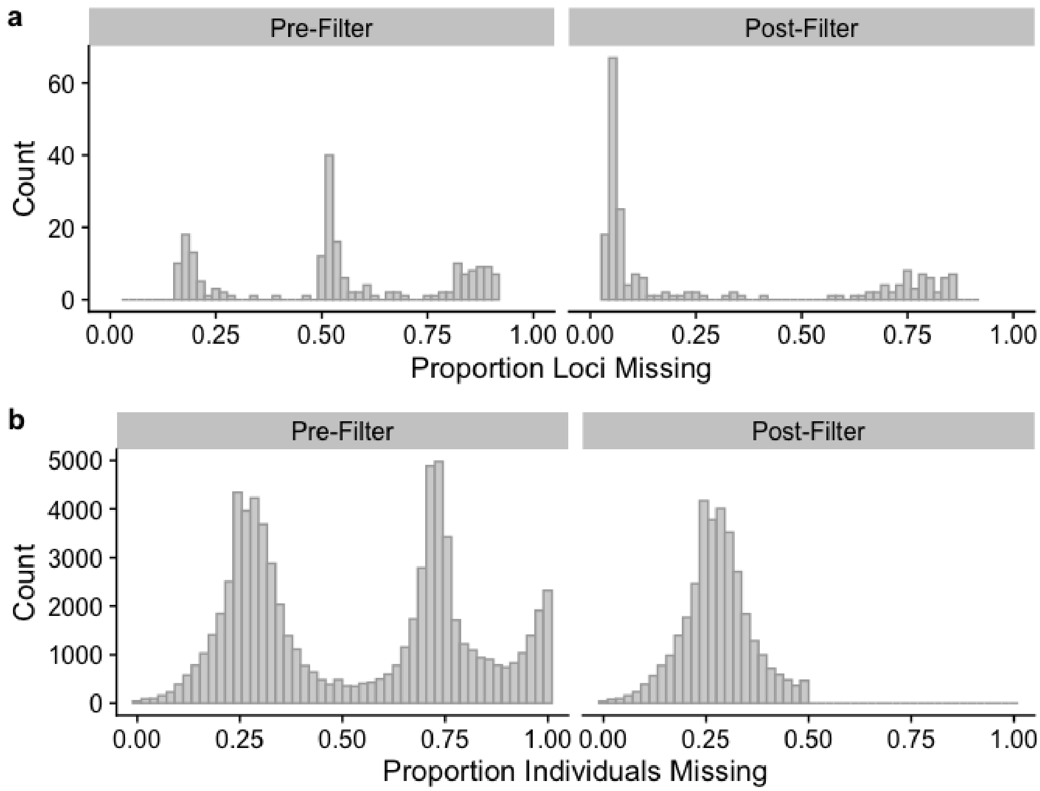

Supplementary Fig. S11.

Distributions of the proportion of missing loci per miracidium (a) or missing miracidia per SNP (b) before (left) and after (right) applying filtering each for excessive missing-ness. Note that sites with low read depth and low genotype quality are coded as missing (see '*Variant set creation*' in Methods) in both pre- and post-filter distributions. Post-filter distributions (right) represent the variant set used for analyses presented here.
